# Transdermal fentanyl induced paralytic intestinal obstruction in advanced liver cancer: a case report

**DOI:** 10.3389/fphar.2025.1550296

**Published:** 2025-03-05

**Authors:** Chen Li, Jindong Chu, Xiaodong Jia, Haibin Su

**Affiliations:** ^1^ Senior Department of Hepatology, The Fifth Medical Center of PLA General Hospital, Beijing, China; ^2^ Senior Department of Oncology, The Fifth Medical Center of PLA General Hospital, Beijing, China

**Keywords:** transdermal fentanyl, intestinal obstruction, adverse drug reactions, liver cancer, pain

## Abstract

**Introduction:**

Transdermal fentanyl (TDF) is a commonly used analgesic drug for managing moderate-to-severe chronic cancer pain. Similar to those observed during the administration of other opioid agonists, the most frequently observed adverse drug reactions during TDF administration include nausea, vomiting, and constipation. However, there have been no reports of TDF causing intestinal obstruction yet. We report a case of TDF-induced paralytic intestinal obstruction confirmed by clinical presentations and imaging findings.

**Case presentation:**

We administered TDF (4.2 mg once every 72 h) for external use to a patient who was admitted with acute upper gastrointestinal bleeding, suffering from advanced liver cancer, and having previously received irregular analgesia. Despite achieving satisfactory analgesic effects, he developed nausea, vomiting, constipation, reduced anal exhaust, and absence of bowel sounds on the fifth day of TDF administration. An X-ray test revealed the presence of flatulence and signs of air fluid levels in the intestine. Conventional treatment was ineffective, and paralytic intestinal obstruction was finally alleviated only after TDF was substituted with oral morphine.

**Conclusion:**

Our findings indicate that, even when TDF is administered in conventional doses, there is a risk of inducing rare cases of intestinal obstruction. In the event of such an occurrence, adjusting the analgesic treatment plan should be the utmost priority.

## Introduction

Transdermal fentanyl (TDF) is a highly effective opioid agonist utilized in the management of moderate-to-severe chronic cancer pain. It exhibits the advantageous qualities of minimal irritability, low allergenicity, excellent viscosity, and effortless peelableity, thereby providing patients with a high level of comfort. Furthermore, TDF eliminates the first-pass effect of the liver, resulting in a substantial enhancement in its bioavailability (Fu et al., 2024). The adverse drug reactions (ADRs) that are commonly associated with TDF are similar to those observed with other opioids and primarily include controllable constipation, nausea, and vomiting (Fu et al., 2024; Gustafsson et al., 2023). Nonetheless, intentional and unintentional misuse, abuse, and overuse, as well as the utilization of TDF in heated environments, can have potentially life-threatening consequences (Nelson and Schwaner, 2009; Carson et al., 2010; Sindali et al., 2012; Bakovic et al., 2015).

At present, no English or Chinese literature reports on TDF-induced paralytic intestinal obstruction exist. We report a patient with advanced liver cancer complicated with acute upper gastrointestinal bleeding (UGIB) who developed paralytic intestinal obstruction during TDF administration in conventional doses. After analgesic medication adjustment and aggressive symptomatic treatment, the patient’s intestinal obstruction was successfully alleviated.

## Case presentation

**FIGURE 1:**
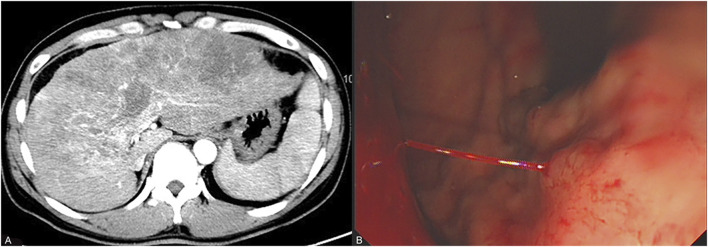
Results of the patient’s enhanced CT scan and gastroscopy. **(A)** represents the enhanced CT showing that the patient is in the stages of cirrhosis and advanced liver cancer 2 months before admission. **(B)** represents the emergency gastroscopy showing that the patient is experiencing acute gastric variceal bleeding 1 day before admission.

A 45-year-old Chinese male was diagnosed with chronic hepatitis B in 2010 but did not receive any treatment. In October 2023, he suffered from upper right abdominal pain, and subsequent laboratory tests revealed HBVDNA levels of 2.54 × 10^4^ IU/mL with a Child–Pugh score of 7 (grade B). Enhanced CT showed cirrhosis, splenomegaly, multiple malignant tumors within the liver, and tumor thrombi in the main portal vein and its left and right branches (Figure 1A). The patient received treatment with sorafenib (0.4 g twice daily) and entecavir (0.5 mg once daily) on 16 November 2023, for advanced liver cancer of Barcelona Clinic Liver Cancer (BCLC) stage C and hepatitis B virus (HBV)–related cirrhosis. As a result of the intermittent and irregular use of ibuprofen sustained-release capsules or tramadol hydrochloride sustained-release tablets for pain relief since December 2023, his visual analogue scale (VAS) score decreased from 4–6 points to 2 points. On 27 December 2023, he vomited 600 mL of blood caused by gastric variceal bleeding (Figure 1B). Subsequently, he underwent tissue adhesive injection under gastroscopy examination for hemostasis treatment. Physical examination showed that his skin and sclera were yellow-stained, he was positive for shifting dullness, his bowel sounds were normal, and he had pitting edema of the lower limbs. His body temperature was 36.3°C, and the indoor temperature was 20.0°C. The patient did not suffer from hypertension, diabetes, cardiovascular and cerebrovascular diseases, chronic hepatitis C, syphilis, or human immunodeficiency virus infection. He had no history of drug abuse or long-term illegal use of opioids. He also had no chronic medication history of anticholinergics, calcium channel blockers, or sedatives. Laboratory tests revealed the alanine aminotransferase value of 89 U/L, aspartate aminotransferase value of 225 U/L, total bilirubin value of 66.7 μmol/L, direct bilirubin value of 53.9 μmol/L, albumin value of 25 g/L, alkaline phosphatase value of 195 U/L, glutamyltransferase value of 347 U/L, international normalized ratio of 1.73, alpha-fetoprotein value of 171 ng/mL, HBVDNA titer of 7.22 × 10^1^ IU/mL, white blood cell count of 11.5 × 10^9^/L, red blood cell (RBC) count of 2.2 × 10^12^/L, hemoglobin level of 73 g/L, and platelet count of 139 × 10^9^/L (Table 1). The patient was diagnosed with decompensated cirrhosis (Child–Pugh score of 11, grade C) accompanied with gastric variceal bleeding, ascites, and advanced liver cancer (BCLC stage D).

**TABLE 1:** The laboratory test results of this patient after admission.

Variables	Result	Normal range
Alanine aminotransferase (U/L)	89	9–50
Aspartate aminotransferase (U/L)	225	15–40
Total bilirubin (µmol/L)	66.7	0–26
Direct bilirubin (µmol/L)	53.9	0–4
Albumin (g/L)	25	40–55
Alkaline phosphatase (U/L)	195	45–125
Glutamyltransferase (U/L)	347	10–60
International normalized ratio	1.73	0.8–1.2
Alpha-fetoprotein (ng/mL)	171	0–7
Des-gamma-carboxy prothrombin (mAU/mL)	5677.24	<40
HBVDNA (IU/mL)	7.22 × 10^1^	<40
White blood cell ( ×10^9^/L)	11.5	3.5–9.5
Red blood cell ( ×10^12^/L)	2.2	4.3–5.8
Hemoglobin (g/L)	73	130–175
Platelet ( ×10^9^/L)	139	125–350
Serum sodium (mmol/L)	134	137–147
Serum potassium (mmol/L)	3.9	3.5–5.3
Hepatitis B surface antigen	+	-
Hepatitis C virus antibody	-	-
Human immunodeficiency virus antibody	-	-
Syphilis antibody	-	-

**FIGURE 2:**
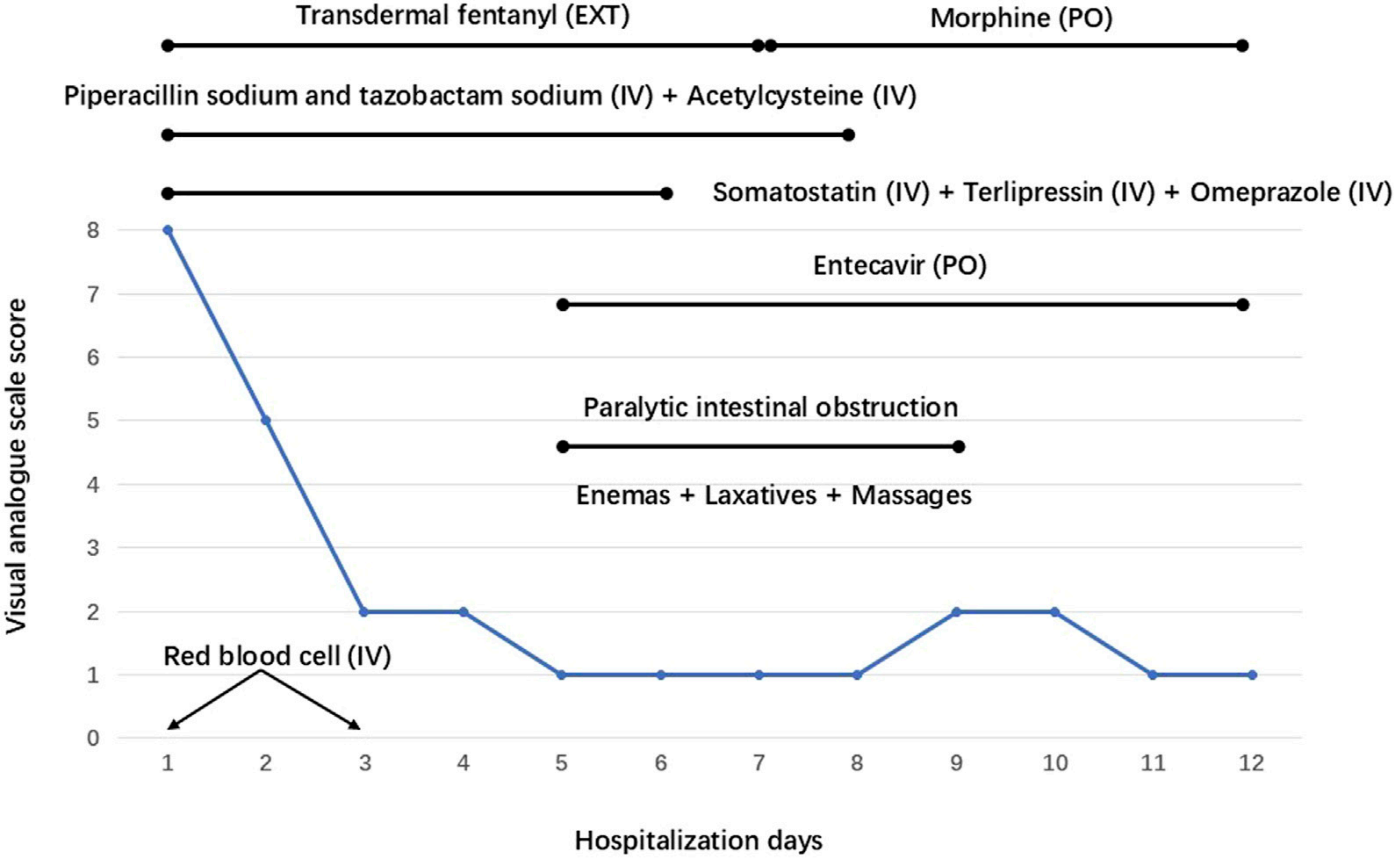
Dynamic trends of the visual analogue scale and treatment measures implemented on the patient during his hospitalization. EXT: External, PO: Per Os, IV: Intravenous.

**FIGURE 3:**
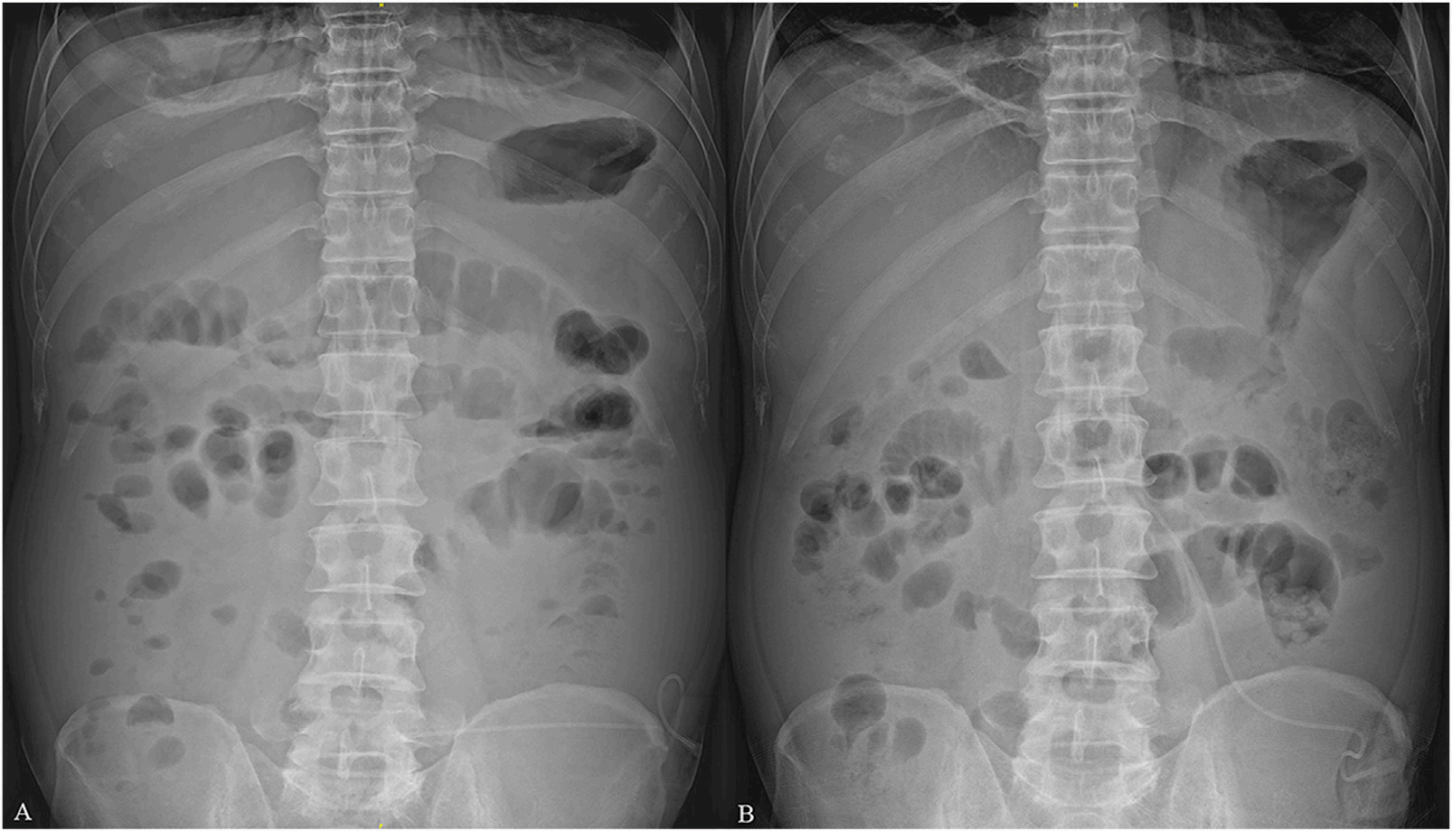
Plain abdominal radiographs of the patient. **(A)** indicates that the patient exhibited signs of intestinal obstruction on the fifth day after admission, including flatulence and air fluid levels, on plain abdominal radiograph after receiving transdermal fentanyl (TDF). **(B)** indicates that the patient’s air liquid level disappeared, and the signs of intestinal obstruction improved considerably after TDF was replaced with oral morphine on the 11th day after admission.

The patient immediately underwent treatment involving fasting and total parenteral nutrition and discontinued sorafenib. He received somatostatin (days 1–6), terlipressin (days 1–6), and omeprazole (days 1–6) for hemostasis; RBC (days 1 and 3) to replenish blood volume; antibiotics (piperacillin sodium and tazobactam sodium, days 1–8) for infection prevention; and acetylcysteine (days 1–8) for hepatocyte protection after admission. Given his persistent severe upper right abdominal pain (VAS score of 8), TDF (4.2 mg once every 72 h) was administered at different sites on his chest on the first and fourth days after admission. This treatment considerably relieved his pain (VAS scores of 1–2) (Figure 2). On the fifth day after admission, his bleeding stopped, and he resumed entecavir for HBV. However, he experienced nausea, vomiting, constipation, reduced anal exhaust, and bowel sounds disappearance. A plain abdominal radiograph indicated the presence of intestinal obstruction (Figure 3A). Despite the administration of enemas, laxatives, and abdominal massages, the therapeutic effect remained unsatisfactory. In suspicion of ADRs stemming from TDF, the medication was discontinued on the seventh day after admission and substituted with morphine sulfate sustained-release tablets (30 mg once daily) for pain management. Two days after switching from TDF to oral morphine, the patient’s discomfort symptoms were effectively alleviated and satisfactory analgesic effects were achieved. On the 11th day after admission, a plain abdominal radiograph revealed remarkable improvement in the patient’s paralytic intestinal obstruction (Figure 3B). Two months after discharge, the patient continued to receive morphine sulfate sustained-release tablets for pain management and exhibited a stable condition with VAS scores of 1–2 and a Child–Pugh score of 9 (grade B). During the follow-up period, he only experienced mild constipation, which was relieved by taking laxatives, and no further intestinal obstruction occurred.

## Discussion

In patients with malignancies, pain represents one of the most prevalent clinical manifestations, substantially affecting quality of life and imposing substantial psychological burden. TDF, a potent opioid analgesic, has gained widespread utilization owing to its convenient application and pronounced therapeutic efficacy worldwide (World Health Organization, 2018). Its key benefits include noninvasive administration, stable blood drug concentration, 3-day medication interval, absorption that is unaffected by gastrointestinal status, and inactive metabolites. Therefore, TDF is suitable for patients with cancer who have stable opioid needs but are not convenient for oral administration and have liver and kidney dysfunction (Fu et al., 2024).

A study involving 459 Chinese patients suffering from moderate-to-severe cancer pain revealed that after using TDF for 2 weeks, their pain intensity notably reduced, with an overall remission rate of 91.29%. Furthermore, their quality of life exhibited considerable enhancement. The most frequently reported ADRs were constipation (14.35%) and nausea (11.39%), whereas only 3.8% of the patients experienced severe adverse events (Zhu et al., 2011). Nine randomized controlled trials demonstrated that compared with oral morphine (46%), TDF (28%) considerably reduced the incidence of constipation in patients with cancer pain (Hadley et al., 2013). The effectiveness of TDF in managing chronic cancer pain, as well as its effect on quality of life, is comparable to that of oral morphine. However, more patients prefer TDF over oral morphine due to its convenience and lower incidence of constipation (Muijsers and Wagstaff, 2001). It is worth noting that excessively high temperatures may increase the permeability of the skin to TDF, thereby posing a life-threatening risk of overdose (Newshan, 1998; Sindali et al., 2012).

Given the patient’s condition, which included advanced liver cancer, decompensated cirrhosis, and acute UGIB, TDF was selected as the analgesic treatment option on the day of hospitalization. However, on the fifth day of using TDF, the patient exhibited typical symptoms of paralytic intestinal obstruction. Somatostatin, terlipressin, antibiotics, omeprazole, entecavir, and acetylcysteine, which were used concurrently with TDF, have not been reported in either their instructions or the literature to have the adverse effect of intestinal obstruction. The instructions for these drugs and previous literature also did not indicate that the combination with TDF increases the risk of intestinal obstruction. The patient also did not experience severe infection, surgical operation, electrolyte imbalance, anticholinergics, or sedatives. Therefore, we believe that TDF, as an opioid, is most likely to induce paralytic intestinal obstruction in this patient. Conventional treatment was ineffective, and discontinuing TDF for relief further confirmed our hypothesis.

The mechanism underlying TDF-induced paralytic intestinal obstruction in this patient may be attributed to the following three factors: First, opioid-induced constipation (OIC). Opioids bind to the μ- and δ-opioid receptors in the intestine, blocking the rhythmic contraction of the intestinal wall and causing slow intestinal peristalsis, reduced intestinal fluid secretion, and increased absorption. Opioids reduce the activity of intestinal muscle plexus neurons, increase the tension of intestinal smooth muscle, and result in the diminished sensitivity of patients to the defecation reflex. In addition, the intestine may develop tolerance to opioids, leading to persistent constipation and triggering intestinal obstruction (Nelson and Camilleri, 2016; Fu et al., 2024). Peripherally acting µ-opioid receptor antagonists (PAMORAs), including methylnaltrexone, naloxegol, naldemedine, and alvimopan, have been proven to improve OIC (Lacy and Cangemi, 2024). Second, opioids induced transient intestinal ischemia. Histopathological analysis has revealed that opioids have the capacity to enhance markedly the thickness of the intestinal submucosa; this effect is characterized by the infiltration of lymphocytes, plasma cells, and an abundance of eosinophils. This process is further accompanied with the dilation of blood vessels and lymphatic channels. Consequently, concentric fibrous thickening and stricture emerge within the intestinal submucosa, ultimately leading to the transient ischemia of the intestine and subsequent fibrosis (Joshi et al., 2018). When opioids are abused, they can induce gastrointestinal ulcers and/or ulcerative strictures, which in turn may result in UGIB and gastrointestinal obstruction (Mahajan et al., 2020). Third, individual factors of this patient may have contributed to TDF-induced paralytic intestinal obstruction. Cirrhosis is prone to constipation due to portal hypertension. Acute UGIB that results in the accumulation of blood in the intestine can potentially exacerbate constipation and induce hepatic encephalopathy. As reported in a study, his previous history of irregular weak opioid (tramadol hydrochloride) treatment may also exacerbate the risk of constipation (Corli et al., 2018).

## Conclusion

TDF stands out as a safe, effective, and convenient opioid analgesic for patients with cancer experiencing moderate-to-severe chronic pain. Constipation, nausea, vomiting, and drowsiness are common ADRs associated with its use. We report a case of TDF-induced paralytic intestinal obstruction in conventional doses, which should be taken seriously by clinical and pharmaceutical experts. For such cases, eliminating triggers; providing enemas, laxatives, and massage therapy; and timely replacement with equivalent analgesics are necessary to avoid serious complications. PAMORAs may also alleviate symptoms of constipation.

## Data Availability

The raw data supporting the conclusions of this article will be made available by the authors, without undue reservation.
